# The Use of Cognitive Screening in Pharmacotherapy Trials for Cognitive Impairment Associated With Schizophrenia

**DOI:** 10.3389/fpsyt.2019.00648

**Published:** 2019-09-06

**Authors:** Jack Cotter, Jennifer H. Barnett, Kiri Granger

**Affiliations:** ^1^Cambridge Cognition, Cambridge, United Kingdom; ^2^Department of Psychiatry, University of Cambridge, Cambridge, United Kingdom

**Keywords:** clinical trial, cognition, pharmacotherapy, psychosis, schizophrenia, trial methodology

## Abstract

There are currently no regulatory approved pharmacological treatments for cognitive impairment associated with schizophrenia (CIAS). One possibility is that trial methodology itself is hindering their development. Emerging evidence suggests that patients with schizophrenia may show limited benefit from pro-cognitive interventions if they already exhibit intact cognitive performance, relative to normative thresholds. The aim of this report was to examine the extent to which objectively assessed cognitive performance has been used as an eligibility and/or stratification criterion in CIAS pharmacotherapy trials. On 16^th^ January 2019, we conducted a systematic search of studies listed on ClinicalTrials.gov to identify randomized, double-blind, placebo-controlled, add-on pharmacotherapy trials conducted in patients with a diagnosis of schizophrenia, in which a paper-and-pencil or computerized cognitive task (or battery) was specified as a primary outcome measure. Of the 87 trials that met our inclusion criteria, 10 (11.5%) required the presence of an objectively assessed cognitive deficit as part of their patient eligibility criteria. No studies reported stratifying patients according to the presence or degree of cognitive impairment they exhibited. These results suggest that the vast majority of CIAS trials may have been underpowered due to the inclusion of cognitively “normal” patients. Purposive screening for cognitive impairment could increase CIAS trial success.

## Introduction

Cognitive impairment is common in people with schizophrenia and is among the strongest predictors of functional disability in this patient group (1–3). Despite considerable efforts and some initially promising results, there are currently no regulatory approved pharmacological treatments for cognitive impairment associated with schizophrenia (CIAS) (4, 5). It remains unclear, however, whether this is truly due to these compounds being ineffective, or whether trial methodology itself has been a limiting factor in successfully demonstrating the efficacy of these agents.

Schizophrenia is a heterogeneous disorder associated with varying clinical and cognitive profiles (6). Though the majority of patients with schizophrenia exhibit some general cognitive dysfunction compared to antecedent expectations, such as premorbid intelligence (7, 8), there is evidence that approximately a quarter of patients display cognitive performance comparable to healthy controls (9–12). Recent evidence has indicated that these “normally” performing patients are significantly less likely to exhibit changes in cognition when participating in CIAS trials (13, 14), suggesting that inclusion of these individuals may limit the scope to detect a pro-cognitive efficacy signal. Contemporary evidence has also reported that there is no association between cognitive performance and measures of social, vocational or everyday functioning in these cognitively “normal” patients (15), suggesting that other illness-related variables are the primary drivers of functional disability among these individuals. Given that cognition is thought to remain relatively stable following the first psychotic episode (11, 16), the rationale for including and administering an investigational medicinal product to this subset of “normally” performing patients is becoming increasingly unclear. Exclusion of these individuals, or at least identifying and ensuring equal stratification of these “normal” performers across trial arms during randomization, may provide additional power to observe pro-cognitive treatment effects, though it is currently unclear whether either of these approaches have been routinely adopted.

The aim of this report was to examine the extent to which objectively assessed cognitive performance has been used as an eligibility and/or stratification criterion in CIAS pharmacotherapy trials.

## Methods

On 16th January 2019, we conducted a systematic search of trials listed on ClinicalTrials.gov (since its inception) using the keyword “schizophrenia” (*n* = 2,978). Eligible studies were those recorded as randomized, double-blind, placebo-controlled, add-on pharmacotherapy trials conducted in patients with a diagnosis of schizophrenia, in which a paper-and-pencil or computerized cognitive task (or battery) was specified as a primary outcome measure. Phase 1 studies (which typically focus on drug safety), nutraceutical interventions (including pharmaceutical-grade dietary supplements) and single-dose studies (including crossover trials) were excluded. We cross-referenced study information listed on ClinicalTrials.gov with corresponding published literature where this could be identified. For all eligible trials, information was manually extracted (where provided) on: i) study details; ii) cognition-related study eligibility criteria; iii) cognition-related stratification procedures or sensitivity analyses.

## Results

Of the 87 trials that met our inclusion criteria, 10 (11.5%) explicitly reported requiring the presence of an objectively measured cognitive deficit as part of their patient eligibility criteria (**Table 1**; **Supplementary Table 1**). In contrast, six trials (6.9%) screened and excluded individuals with very high cognitive performance in an effort to avoid ceiling effects, while the majority of trials excluded those with (or at increased risk of) severe cognitive impairments that could potentially confound cognitive testing, such as low IQ, neurological or developmental disorders or a history of head trauma. A number of the included studies also stratified patients during randomization according to factors that could potentially influence treatment response, such as their sex, recruiting site, genetic profile, tobacco use, or inpatient/outpatient status. However, we found no studies reporting the stratification of patients according to the presence or degree of cognitive impairment exhibited at screening or baseline. Similarly, we identified only a single study that listed an intention to perform post-trial sensitivity analyses to establish whether any pro-cognitive effects were exclusive to those exhibiting cognitive impairment at baseline.

**Table 1:** Eligible CIAS pharmacotherapy trials that listed an objectively measured cognitive deficit among their patient inclusion criteria.

Trial phase (as listed on ClinicalTrials.gov)	Total	Phase 2	Phase 2/3	Phase 3	Phase 4	Not reported

Number of eligible trials identified	87	46	2	9	19	11
Number requiring objectively assessed cognitive deficit for patient inclusion (%)	10 (11.5%)	6 (13%)	0 (0%)	0 (0%)	3 (15.8%)	1 (9.1%)

## Discussion

The vast majority of CIAS pharmacotherapy trials have not included formal eligibility criteria to ensure participants exhibit a cognitive deficit as part of their study screening procedures. While this is consistent with consensus guidelines that have previously recommended such an approach (4), the failure to develop any effective compounds, coupled with recent evidence suggesting that the inclusion of “normal” performers may limit the ability to detect pro-cognitive effects, has brought the rationale for this into question.

In the absence of purposive screening for cognitive impairment, an important next step is to clarify how many patients entering CIAS trials already perform within the limits of “normal” cognitive performance. Pooled cognitive data from 15 multi-site trials involving patients with schizophrenia (*n* = 2,616) has recently been published, including both composite and domain-specific baseline MATRICS Consensus Cognitive Battery (MCCB) T scores (17). These T scores are standardized to age and sex-matched normative data, and have an estimated mean of 50 and standard deviation of 10 in the general healthy population (18). While the individual patient-level data is unavailable, visual inspection of the associated histograms suggests MCCB scores from the pooled trial data were broadly normally distributed (17). This coupled with the published sample means and standard deviations allow inferences to be made regarding the proportion of patients that performed within different cut-offs of the healthy normative mean. On this basis, it appears that a number of patients scored within the normative range across each of the cognitive domains, for example, approximately fifty percent of patients had a T score equal to or greater than 40 for the “problem solving” MCCB subscale. Though the trials included in these pooled analyses were not exclusively pro-cognitive pharmacotherapy trials, the patient eligibility criteria used across these studies reflected those typically used in CIAS trials. Further analyses of existing patient-level data would help to clarify what proportion of patients entering CIAS pharmacotherapy trials already exhibit cognitive performance comparable to healthy controls. This will be valuable in helping to guide decision making in future CIAS trial design.

For the purposes of demonstrating efficacy, pro-cognitive intervention trials should be powered to detect a “clinically meaningful” difference between treatment groups, typically equating to an effect size in the small-medium range (e.g., Cohen’s d ≥ 0.3). However, recent evidence has suggested that it is unlikely that one would expect to see gains in cognition this large in up to a quarter of patients who score similarly to healthy controls on cognitive tasks (9–13). This means that the poorer performing patients would need to exhibit substantially greater cognitive gains in order to meet the pre-specified average group-based level of improvement. Based on the results of our database search, it also indicates that the majority of CIAS trials have likely been underpowered to detect beneficial treatment effects.

An interesting parallel in schizophrenia-related clinical trial methodology can be drawn with the development of treatments for negative symptoms. Like cognitive impairment, these are a common and highly debilitating aspect of schizophrenia for which there are currently no regulatory approved treatments (19–21). However, at the time of writing, all ongoing phase 2 and 3 trials listed on ClinicalTrials.gov in which a negative symptom measure is specified as the primary outcome, require that patients exhibit at least a moderate level of related symptomatology at screening and at baseline in order to be eligible for inclusion.

While it is more commercially practical and appealing to develop a treatment aimed at all patients within a diagnostic category, the substantial development costs involved, ever-increasing unmet need for an effective CIAS treatment and growing movement towards precision psychiatry (matching the right drug to the right patient at the right time) may make cognitive screening in the context of CIAS trials a necessity. There is also an argument that exposing cognitively normal patients to these investigational drugs represents an unnecessary and unethical risk, particularly given that polypharmacy is already common among patients with schizophrenia (22). Cognitive screening has already been successfully adopted in a small number of CIAS trials and could be adapted for use in clinical practice (if necessary) using relatively simple and brief testing procedures as is routine in other indications, such as Alzheimer’s disease.

Practical considerations include the need to determine a consensus on suitable measures and thresholds for establishing a relevant level of cognitive impairment among patients with schizophrenia. Trials identified in our search used a range of tools and criteria to identify “impaired” patients (**Supplementary Table 1**), though a threshold ≥ 1 standard deviation below the healthy normative mean on an objective cognitive task(s) would align with similar guidelines in other disease-areas, as well as recent studies investigating the impact of cognitive impairment on real-world functioning in schizophrenia (15, 23). There is also an argument that this should be based on the hypothesized mechanism of action of the study drug and the cognitive domains thought most likely to be enhanced. Whether to then exclude or stratify “normally” performing patients across study arms during randomization are also potentially important considerations in this field, while pre-planned subgroup analyses may provide a practical intermediate approach, particularly during early stages of drug development.

Though the focus of this article is on CIAS pharmacotherapy trials, cognitive screening in the context of pro-cognitive intervention studies is likely to have implications beyond schizophrenia. Cognitive dysfunction is an important target for therapeutic intervention across a range of psychiatric populations, though substantial heterogeneity is also evident in cognitive profiles within these diagnostic groups (24–26). *Post hoc* analyses of trials involving patients with mood disorders also suggest that individuals with an objectively assessed cognitive deficit at baseline are substantially more likely to exhibit a clinically relevant improvement post-treatment, relative to those with normal cognitive functioning (27). This has led to similar calls for the use of cognitive screening in clinical trials to ensure the inclusion of only those patients with a clinically relevant deficit (23).

For the purposes of this report, we used ClinicalTrials.gov to identify randomized, double-blind, placebo-controlled, add-on pharmacotherapy trials conducted in patients with a diagnosis of schizophrenia, in which a paper-and-pencil or computerized cognitive task (or battery) was specified as a primary outcome measure. These study designs are widely recognized as the “gold standard” for evidence generation and are recommended by the National Institute of Mental Health (NIMH) MATRICS panel for use in CIAS trials (28). While this may not have provided an exhaustive list of all CIAS trials that have been conducted, it does provide a useful snapshot of contemporary methodological practices in this field, particularly for major commercial studies. We attempted to identify corresponding published literature for all trials in an effort to verify details listed on ClinicalTrials.gov (further information was found for 41 (47.1%) of the included trials). We excluded Phase 1 trials, which typically focus on establishing the safety of a compound while looking at potential efficacy signals across a range of clinical and symptomatic measures, though screening for cognitive dysfunction among patients included in these early phase studies may also be useful when attempting to detect a hypothesized pro-cognitive effect of a novel compound.

In conclusion, this paper highlights that purposive screening for cognitive impairment among patients with schizophrenia could increase statistical power to detect pro-cognitive treatment effects in clinical trials. While the focus of this article has been on pharmacotherapy studies, this could also have implications for other forms of therapeutic interventions. Further *post hoc* analyses of completed trials could help to shed further light on this hypothesis and ultimately aid in the development of effective treatments for this area of significant unmet clinical need.

## Author Contributions

JC designed and performed the database search and drafted the manuscript. JB and KG critically reviewed and approved the manuscript prior to its submission for publication.

## Funding

This research did not receive any specific grant from funding agencies in the public, commercial, or not-for-profit sectors.

## Conflict of Interest Statement

The authors are employees of Cambridge Cognition Ltd.

The reviewer JF declared a past co-authorship with one of the authors JC to the handling editor.
